# Evaluation of classical clinical endpoints as surrogates for overall survival in patients treated with immune checkpoint blockers: a systematic review and meta-analysis

**DOI:** 10.1007/s00432-018-2738-x

**Published:** 2018-08-21

**Authors:** Howard L. Kaufman, Lawrence H. Schwartz, William N. William, Mario Sznol, Kyle Fahrbach, Yingxin Xu, Eric Masson, Andrea Vergara-Silva

**Affiliations:** 1 Massachusetts General Hospital, 55 Fruit St Gray 730, Boston, MA USA; 2 Columbia University College of Physicians and Surgeons, New York Presbyterian Hospital, 622 W 168th St, New York, NY USA; 3 MD Anderson Cancer Center, 1515 Holcombe Blvd, Houston, TX USA; 4 Centro Oncológico BP, a Beneficência Portuguesa de São Paulo, São Paulo, Brazil; 5 Yale School of Medicine, 333 Cedar St, New Haven, CT USA; 6 Evidera, 7101 Wisconsin Ave, Suite 1400, Bethesda, MD USA; 7 AstraZeneca, 35 Gatehouse Dr, Waltham, MA USA; 8 AstraZeneca, One MedImmune Way, Gaithersburg, MD USA; 9 Present Address: Replimune Inc., 18 Commerce Way, Woburn, MA 01801 USA; 10 Present Address: Regeneron Pharmaceuticals Inc., 777 Old Saw Mill River Rd, Tarrytown, NY USA; 11 Present Address: Biogen, 225 Binney St, Cambridge, MA USA; 12 Present Address: Ayala Pharmaceuticals, 1313 N. Market Str, Suite 5100, Wilmington, DE USA

**Keywords:** Immune checkpoint blockers, Surrogate endpoint, Solid tumors, Programmed cell death-1, Programmed cell death ligand-1, Cytotoxic T-lymphocyte-associated antigen-4

## Abstract

**Purpose:**

Classical clinical endpoints [e.g., objective response rate (ORR), disease control rate (DCR), and progression-free survival (PFS)] may not be appropriate for immune checkpoint blockers (ICBs). We evaluated correlations between these endpoints and overall survival (OS) for surrogacy.

**Methods:**

Randomized controlled trials (RCTs) of solid tumors patients treated with ICBs published between 01/2005 and 03/2017, and congress proceedings (2014–2016) were included. Arm-level analyses measured 6-month PFS rate to predict 18-month OS rate. Comparison-level analyses measured ORR odds ratio (OR), DCR OR, and 6-month PFS hazard ratio (HR) to predict OS HR. A pooled analysis for single-agent ICBs and ICBs plus chemotherapy vs chemotherapy was conducted. Studies of single-agent ICBs vs chemotherapy were separately analyzed.

**Results:**

27 RCTs involving 61 treatment arms and 10,300 patients were included. Arm-level analysis showed higher 6- or 9-month PFS rates predicted better 18-month OS rates for ICB arms and/or chemotherapy arms. ICB arms had a higher average OS rate vs chemotherapy for all PFS rates. Comparison-level analysis showed a nonsignificant/weak correlation between ORR OR (adjusted *R*^2^ = − 0.069; *P* = 0.866) or DCR OR (adjusted *R*^2^ = 0.271; *P* = 0.107) and OS HR. PFS HR correlated weakly with OS HR in the pooled (adjusted *R*^2^ = 0.366; *P* = 0.005) and single-agent (adjusted *R*^2^ = 0.452; *P* = 0.005) ICB studies. Six-month PFS HR was highly predictive of OS HR for single-agent ICBs (adjusted *R*^2^ = 0.907; *P* < 0.001), but weakly predictive in the pooled analysis (adjusted *R*^2^ = 0.333; *P* = 0.023).

**Conclusions:**

PFS was an imperfect surrogate for OS. Predictive value of 6-month PFS HR for OS HR in the single-agent ICB analysis requires further exploration.

## Background

Immune checkpoint blockers (ICBs), and specifically the use of antibodies against programmed cell death-1 (PD-1), programmed cell death ligand-1 (PD-L1), and cytotoxic T-lymphocyte-associated antigen-4 (CTLA-4) have been approved for use in several cancer types and demonstrated improved overall survival (OS) compared with the standard therapy (AstraZeneca Pharmaceuticals 2017; Bristol-Myers Squibb 2013; Bristol-Myers Squibb 2017; Genentech 2017; Merck and Company Inc. 2017; Pfizer 2017). The US Food and Drug Administration (FDA) supports the use of surrogate endpoints in oncology trials, especially for accelerated approvals (Johnson et al. 2003). In fact, several chemotherapies have relied on response endpoints, such as objective response rate (ORR) with or without duration data, for the basis of regular approvals (Johnson et al. 2003).

Although improvement in OS is generally the most desirable endpoint in oncology clinical trials, there is an ongoing interest in identifying and validating surrogate endpoints that can better predict the likelihood of OS to improve the design of clinical studies and potentially expedite the approval of novel agents (Booth and Eisenhauer 2012; Foster et al. 2011; Kemp and Prasad 2017). Correlations between endpoints such as progression-free survival (PFS), ORR, disease control rate (DCR), and time to progression and OS have been investigated, but correlations between these endpoints and OS have not been thoroughly investigated for ICBs (Flaherty et al. 2014; Prasad et al. 2015).

Endpoints such as ORR and PFS traditionally used to evaluate the effect of drugs that act directly on the tumor may not be the most appropriate for ICBs that are characterized by a very different mechanism of action. Unlike other cancer therapies that act directly on tumor cells, ICBs act indirectly by enhancing antitumor immune responses and eliciting lymphocyte infiltration into the tumor, thereby frequently resulting in an initial tumor enlargement of varying degrees depending on the tumor type, and possibly the appearance of new lesions, with subsequent reduction in tumor size and number of lesions mediated by ongoing immunologic mechanisms (Hersh et al. 2011; Hodi et al. 2016b; Seymour et al. 2017; Wolchok et al. 2009).

Among several randomized trials investigating ICBs vs standard therapies in patients with non-small-cell lung cancer (NSCLC), renal-cell carcinoma (RCC), and head and neck squamous-cell carcinoma (HNSCC) using the conventional response assessment tool, Response Evaluation Criteria in Solid Tumors (RECIST), it was found that, whereas PFS was similar between ICBs and standard treatment, OS associated with ICBs was statistically superior (Borghaei et al. 2015; Ferris et al. 2016; Herbst et al. 2016; Motzer et al. 2015; Rittmeyer et al. 2017). Only a few randomized trials testing ICBs vs chemotherapy in patients with melanoma and NSCLC have shown an OS benefit associated with ICBs in conjunction with both ORR and PFS benefit, as evaluated by standard RECIST criteria (Reck et al. 2016; Robert et al. 2015a). Furthermore, in a study investigating pembrolizumab in patients with melanoma, response patterns were evaluated using both RECIST and an alternative response assessment tool, called immune-related response criteria (irRC) (Hodi et al. 2016b). RECIST did not appear to capture the true benefit associated with ICBs, and it was suggested that use of irRC may prevent premature discontinuation of ICB therapy.

Relationships between clinical endpoints may vary with the use of single-agent ICBs or combination ICBs, and with the tumor type being treated. For example, in a recent study conducted in patients with RCC treated with ipilimumab/nivolumab combination therapy vs sunitinib, the combination regimen provided a superior OS benefit, but failed to provide a statistically significant PFS benefit over targeted therapy in either the intent to treat (ITT) population or the patients with intermediate/poor risk. Although the PFS curves started to diverge after the 6-month timepoint, in favor of ipilimumab/nivolumab, this trend was not statistically significant (Escudier et al. 2017). In contrast, in a study conducted in patients with NSCLC with a high PD-L1 expression level (tumor proportion score ≥ 50%) treated with pembrolizumab monotherapy vs chemotherapy, the single-agent ICB provided both PFS and OS benefit over chemotherapy (Reck et al. 2016).

The aim of this systematic literature review and meta-analysis was to assess whether any of these endpoints that are typically used in cancer studies could function as surrogates for OS in studies involving ICBs. We identified relevant randomized controlled trials (RCTs) of ICBs over the past 12 years and analyzed both arm- and comparison-level data to explore the relationship between OS and clinical endpoints (ORR, DCR, and PFS) in patients with solid tumors treated with single-agent ICBs or ICBs in combination with chemotherapy, compared with patients treated with chemotherapy.

## Methods

### Literature selection

A systematic literature review was conducted, using Medline, Embase, and CENTRAL (indexed databases) to identify RCTs published between January 2005 and March 2017. Congress proceedings from the American Society of Clinical Oncology, the European Society for Medical Oncology, the American Head and Neck Society, the European Lung Cancer Conference, and the Society for Melanoma Research published between 2014 and 2016 were also searched, as well as 2 clinical trials registries (clinicaltrials.gov and clinicaltrialsregister.eu). Studies that assessed the efficacy of agents targeting PD-1 (nivolumab, pembrolizumab, pidilizumab, MEDI0680, REGN2810, PDR001), PD-L1 (atezolizumab, avelumab, durvalumab), or CTLA-4 (ipilimumab, tremelimumab) in adult patients with melanoma, NSCLC, HNSCC, RCC, or urothelial carcinoma (UC) were selected. The detailed search strategies for this analysis are included in Tables 1 and 2.

**Table 1:** Search terms and yield

	Search criteria	Search algorithm	Search yield (*n*)
EMBASE via OVID			
1	PD-1 inhibitors	(PD-1 inhibit$ OR programmed cell death protein 1 inhibit$ OR anti-programmed death OR antiprogrammed death OR Anti-PD1 OR nivolumab OR Opdivo OR bms-936558 OR bms 936558 OR bms936558 OR mdx-1106 OR mdx 1106 OR mdx1106 OR ono-4538 OR ono 4538 OR ono4538 OR pembrolizumab OR Keytruda OR lambrolizumab OR mk-3475 OR mk 3475 OR mk3475 OR Pidilizumab OR ct 011 OR ct-011 OR ct011 OR Medi0680 OR REGN2810 OR PDR001).ti,ab,tn	2515
2		Nivolumab/OR pembrolizumab/OR pidilizumab/OR programmed death 1 receptor inhibitor/dt [Drug Therapy]	2491
3	PD-L1 inhibitors	(PD-L1 inhibit$ OR programmed death ligand 1 inhibit$ OR anti-programmed death ligand 1 OR antiprogrammed death ligand 1 OR Anti-PD-L1 OR Atezolizumab OR tecentriq OR mpdl-3280a OR mpdl 3280a OR mpdl3280a OR rg-7446 OR rg 7446 OR rg7446 OR Avelumab OR msb-0010718c OR msb 0010718c OR msb0010718c OR Durvalumab OR med-4736 OR med 4736 OR med4736).ti,ab,tn	1086
4		Atezolizumab/OR avelumab/OR durvalumab/OR programmed death 1 ligand 1/dt [Drug therapy]	583
5	CTLA-4 inhibitors	(CTLA-4 inhibit$ OR cytotoxic T-lymphocyte antigen 4 inhibit$ OR Anti-Cytotoxic T-lymphocyte antigen 4 OR anti-CLTA4 OR anti-ctla-4 OR Ipilimumab OR yervoy OR strentarga OR bms-734016 OR bms 734016 OR bms734016 OR mdx-010 OR mdx 010 OR mdx010 OR mdx-101 OR mdx 101 OR mdx101 OR Tremelimumab OR Ticilimumab OR cp-675 206 OR cp 675 206 OR cp675206 OR cp675 206).ti,ab,tn	3084
6		Ipilimumab/OR ticilimumab/OR cytotoxic T-lymphocyte antigen 4 inhibitor/dt [Drug Therapy]	3394
7		1 OR 2 OR 3 OR 4 OR 5 OR 6	6861
8		((Exp animal/or nonhuman/) not exp human/)	
9		7 NOT 8	6312
10		(Book or book series or conference abstract or conference paper or conference proceeding or “conference review” or editorial or letter or note or “review”).pt	
11		9 NOT 10	3099
12		Limit 11 to (english language and year = “2005-Current”)	3090
13		Remove duplicates from 12	2568
MEDLINE via PubMed			
1	PD-1 inhibitors	Programmed cell death 1 receptor/antagonists and inhibitors [mesh] OR PD-1 inhibit* [tiab] OR programmed cell death protein 1 inhibit* [tiab] OR anti-programmed death [tiab] OR antiprogrammed death [tiab] OR Anti-PD1 [tiab] OR nivolumab OR Opdivo OR bms-936558 OR “bms 936558” OR bms936558 OR mdx-1106 OR “mdx 1106” OR mdx1106 OR “ono-4538” OR “ono 4538” OR ono4538 OR pembrolizumab OR Keytruda OR lambrolizumab OR mk-3475 OR “mk 3475” OR mk3475 OR Pidilizumab OR “ct 011” OR ct-011 OR ct011 OR Medi0680 OR REGN2810 OR PDR001	2074
2	PD-L1 inhibitors	Antigens, CD274/antagonists and inhibitors [Mesh] OR PD-L1 inhibit* [tiab] OR programmed death ligand 1 inhibit* [tiab] OR anti-programmed death ligand 1 [tiab] OR antiprogrammed death ligand 1 [tiab] OR Anti-PD-L1 [tiab] OR Atezolizumab OR tecentriq OR mpdl-3280a OR “mpdl 3280a” OR mpdl3280a OR rg-7446 OR “rg 7446” [tiab] OR rg7446 OR Avelumab OR “msb-0010718c” [tiab] OR “msb 0010718c” [tiab] OR “msb0010718c” [tiab] OR Durvalumab OR med-4736 OR “med 4736” OR med4736	888
3	CTLA-4 inhibitors	CTLA-4 Antigen/antagonists and inhibitors [Mesh] OR CTLA-4 inhibit* [tiab] OR cytotoxic T-lymphocyte antigen 4 inhibit* [tiab] OR Anti-Cytotoxic T-lymphocyte antigen 4 [tiab] OR anti-CLTA4 [tiab] OR anti-ctla-4 [tiab] OR Ipilimumab OR yervoy OR strentarga [tiab] OR bms-734016 OR “bms 734016” OR “bms734016” OR mdx-010 OR “mdx 010” OR mdx010 OR mdx-101 OR “mdx 101” OR mdx101 OR Tremelimumab OR Ticilimumab OR “cp-675 206” [tiab] OR “cp 675 206” [tiab] OR “cp675206” OR “cp675 206” [tiab]	2363
4		1 OR 2 OR 3	4214
5		(Review[pt] OR review[ti] OR comment[pt] OR editorial[pt] OR meta-analysis[pt] OR meta-analysis[ti] OR letter[pt] OR in vitro techniques[mh] OR guideline[pt] OR case reports[pt] OR case report[ti] OR news[pt] NOT (((review[pt] OR review[ti] OR letter[pt] OR comment[pt] OR editorial [pt] OR meta-analysis[pt] OR meta-analysis[ti] OR in vitro techniques[mh] OR guideline[pt] OR news[pt] OR case reports[pt]) AND (clinical trial[pt] OR comparative study[pt] OR multicenter study[pt] OR validation studies[pt] OR cohort studies[mh] OR cross-over studies[mh] OR case–control studies[mh] OR follow-up studies[mh] OR cross-sectional studies[mh])) OR (case reports[pt] AND series[tiab])))	2,397,171
6		4 NOT 5	2101
7		(Animals[mh] NOT humans[mh])	1,317,240
8		6 NOT 7	1832
9		Limited to 2005-present and English	1568
Cochrane Central Register of Controlled Trials (CENTRAL)			
1	PD-1 inhibitors	PD-1 inhibit* or programmed cell death protein 1 inhibit* or anti-programmed death or antiprogrammed death or Anti-PD1 or nivolumab or Opdivo or bms-936558 or “bms 936558” or bms936558 or mdx-1106 or “mdx 1106” or mdx1106 or “ono-4538” or “ono 4538” or ono4538 or pembrolizumab or Keytruda or lambrolizumab or mk-3475 or “mk 3475” or mk3475 or Pidilizumab or “ct 011” or ct-011 or ct011 or Medi0680 or REGN2810 or PDR001:ti,ab,kw (Word variations have been searched)	978
2		MeSH descriptor: [Programmed Cell Death 1 Receptor] explode all trees and with qualifier(s): [Antagonists & inhibitors - AI]	10
3	PD-L1 inhibitors	PD-L1 inhibit* or programmed death ligand 1 inhibit* or anti-programmed death ligand 1 or antiprogrammed death ligand 1 or Anti-PD-L1 or Atezolizumab or tecentriq or mpdl-3280a or “mpdl 3280a” or mpdl3280a or rg-7446 or “rg 7446” or rg7446 or Avelumab or “msb-0010718c” or “msb 0010718c” or “msb0010718c” or Durvalumab or med-4736 or “med 4736” or med4736:ti,ab,kw (Word variations have been searched)	454
4		MeSH descriptor: [Antigens, CD274] explode all trees and with qualifier(s): [Antagonists & inhibitors - AI]	7
5	CTLA-4 inhibitors	CTLA-4 inhibit* or cytotoxic T-lymphocyte antigen 4 inhibit* or Anti-Cytotoxic T-lymphocyte antigen 4 or anti-CLTA4 or anti-ctla-4 or Ipilimumab or yervoy or strentarga or bms-734016 or “bms 734016” or “bms734016” or mdx-010 or “mdx 010” or mdx010 or mdx-101 or “mdx 101” or mdx101 or Tremelimumab or Ticilimumab or “cp-675 206” or “cp 675 206” or “cp675206” or “cp675 206”:ti,ab,kw (Word variations have been searched)	625
6		MeSH descriptor: [CTLA-4 Antigen] explode all trees and with qualifier(s): [Antagonists & inhibitors - AI]	4
7		#1 OR #2 OR #3 OR #4 OR #5 OR #6	1410
8		#7 not (pubmed or embase):an	40
9		Limited to 2005-present	30
Clinicaltrials.gov (National Institutes of Health, United States)			
1	PD-1 Inhibitors	On February 7, 2017. clinicaltrials.gov was searched by clinical trial identifier numbers that were obtained from the literature search. The following values were searched “NCT01927419”, “NCT01783938”, “NCT01704287”, “NCT01721772”, “NCT01866319”, “NCT01721746”, “NCT02039674”, “NCT01905657”, “NCT01642004”, “NCT01673867”, “NCT02041533”, “NCT02142738”, “NCT01928394”, “NCT02256436”, “NCT01354431”, “NCT02105636”	16
2	PD-L1 inhibitors	“NCT01903993”, “NCT02008227”	2
3	CTLA-4 inhibitors	“NCT01927419”, “NCT00289640”, “NCT00162123”, “NCT00261365”, “NCT01783938”, “NCT00050102”, “NCT01134614”, “NCT01740297”, “NCT00257205”, “NCT00324155”, “NCT00527735”, “NCT01928394”	12
4		#1 OR #2 OR #3	27
Clinicaltrialsregister.eu (European Union or European Economic Area)			
1	PD-1 inhibitors	(“nivolumab OR pembrolizumab OR pidilizumab”)	182
2	PD-L1 inhibitors	(“atezolizumab OR avelumab OR durvalumab”)	92
3	CTLA-4 inhibitors	(“ipilimumab OR ticilimumab OR tremelimumab”)	124
4		#1 OR #2 OR #3	332

Search strategies used to identify relevant citations in indexed databases (EMBASE/MEDLINE/CENTRAL) and in clinical trial registries

**Table 2:** Conference proceedings’ search strategy

Conference	Strategy
ASCO	We used the ASCO Website—http://meetinglibrary.asco.org/abstracts—to screen abstracts from the annual meetings (2014, 2015, and 2016) using labels/terms for the 3 indications of interest, and combined that with terms for immunotherapy agent classes, respectively Non-small-cell lung cancer, lung non-small-cell cancer, large cell carcinoma Melanoma, melanoma skin cancer Head and neck cancer, laryngectomy, oral cancer, oral neoplasm, oral tumor Cancer immunotherapy, immunotherapy, PD-1, PD-L1, CTLA-4
ESMO	We used the ESMO Website—http://www.esmo.org/Conferences/Past-Conferences—to search for abstracts presented at ESMO 2014, 2015, and 2016. The search results were refined by the following topics (as available in each conference) Non-small-cell lung cancer, lung non-small-cell cancer, large cell carcinoma Melanoma, melanoma skin cancer Head and neck cancer, laryngectomy, oral cancer, oral neoplasm, oral tumor The 3 topics above were combined, respectively, with terms for cancer immunotherapy in the “abstract” section Cancer immunotherapy, immunotherapy, PD-1, PD-L1, CTLA-4
AHNS	We used the AHNS Website—https://www.ahns.info/ahns-previous-meetings/—to screen all oral and poster presentations accepted for 2014, 2015, and 2016 annual conferences, using the terms under Head and neck cancer, laryngectomy, oral cancer, oral neoplasm, oral tumor Cancer immunotherapy, immunotherapy, PD-1, PD-L1, CTLA-4
ESMO/ELCC^a^	All 2014 ELCC-accepted abstracts were published as a supplement of the *Journal of Thoracic Oncology*. We used the journal’s Website—http://www.jto.org/article/S1556-0864(15)30267-7/pdf—to screen all 2014 ELCC abstracts and posters using the terms under Non-small-cell lung cancer, lung non-small-cell cancer, large cell carcinoma Melanoma, melanoma skin cancer Cancer immunotherapy, immunotherapy, PD-1, PD-L1, CTLA-4
SMR^a^	We used the SMR Website—https://www.societymelanomaresearch.org/meetings/past—to screen abstracts from the 2015 and 2016 annual meetings using labels/terms for Melanoma, melanoma skin cancer Cancer immunotherapy, immunotherapy, PD-1, PD-L1, CTLA-4

Search strategies used to identify relevant citations in Conference Proceedings

*AHNS* American Head and Neck Society, *ASCO* American Society of Clinical Oncology, *CTLA-4* cytotoxic T-lymphocyte-associated antigen-4, *ELCC* European Lung Cancer Conference, *ESMO* European Society for Medical Oncology, *PD-1* programmed cell death-1, *PD-L1* programmed cell death ligand-1, *SMR* Society for Melanoma Research

^a^Several conferences, such as ELCC 2015, ELCC 2016, and SMR 2014, are not searchable via publicly available resources or websites

Publications were initially screened by title and abstract by a single investigator with 10% of the selections reviewed by a second investigator and discrepancies resolved by consensus, or by a third investigator. Once selected as relevant, the full-text articles of the publications were reviewed. For inclusion in this analysis, the study had to report OS in addition to at least one other clinical endpoint [i.e., PFS, ORR, and DCR (complete response + partial response + stable disease), per RECIST or modified World Health Organization (WHO) criteria] as determined by review of the full article.

Studies were excluded if the investigation focused on another class of immunotherapy, such as a vaccine or cytokine-based agents, or if the ICB was delivered concurrently with radiotherapy and/or surgery, or if non-pharmacologic interventions were used as comparators. To minimize risk of bias, case studies, case series, and case reports were excluded from the analysis in favor of RCTs.

### Data source

In the arm-level analysis, studies were included if each treatment arm’s absolute effects were reported/could be derived for ORR, DCR, 6- and 9-month PFS, median PFS, median OS, or OS at 12 or 18 months. For the comparison-level analyses, studies were included if the treatments’ relative effects [odds ratios (ORs)] on ORR and DCR or hazard ratios (HRs) on PFS and OS were reported/could be derived. When HRs for PFS/OS or PFS/OS rates at specific timepoints were unavailable, the Kaplan–Meier graphs were digitized to manually calculate this information (Guyot et al. 2012; Hoyle and Henley 2011). Similarly, when ORs for ORR/DCR were unavailable, these values were similarly calculated.

Relevant data were directly extracted from studies as reported. Arm-level data for each of the outcomes were extracted including the number of patients with the event, number of patients evaluated for the event, and the ITT or modified ITT results. Comparison-level data comparing the 2 treatments on any of the outcomes were also extracted including relative risk (OR or HR).

Data from full publications were extracted by one investigator. Data presented at congresses for the same study were reviewed and any unique, additional data were identified and captured. Data extraction was independently validated by a second investigator, and a third investigator was consulted to resolve disagreements, if necessary.

Arm-level correlative analysis provides initial insights into the absolute effect of a therapy on any given endpoint, although this analysis is limited by the inherent association between the selected candidate surrogates and OS in the same treatment arm, and is likely to be confounded by variations in baseline patient characteristics across different studies (Prasad et al. 2015). For example, a study with a patient population with poorer performance status is more likely to have both lower PFS and OS at any given timepoint than a study with healthier patients. Thus, a strong correlation identified by arm-level analysis can be an artifact, to some degree, of differences in patient populations across studies. Correlations found with arm-level analysis are less reliable compared with correlations found using data from multiple trials (comparison-level/trial-level correlative analysis) (Prasad et al. 2015). Comparison-level/trial-level correlative analysis provides insights into the relative effect of a therapy on a given endpoint, establishing the most reliable surrogates (Prasad et al. 2015). However, if a variable is a treatment-effect modifier, such that the relative effect is greatly dependent on factors that vary across studies, findings obtained with comparison-level analysis may not be reliable or generalizable.

### Statistical analysis

A pooled analysis was conducted for single-agent ICBs and ICBs in combination with chemotherapy vs chemotherapy. A separate analysis was also conducted for studies including only ICBs as single agents vs chemotherapy. Weighted linear regression models were fitted with adjusted *R*^2^ values calculated to estimate the total amount of variation explainable by the predictor. Unlike standard *R*^2^ values, the adjusted *R*^2^ values account for the number of predictors in the model, and the closer the adjusted *R*^2^ is to 1, the stronger the correlation. We stratified analyses by treatment regimen (single-agent ICB or ICB in combination with chemotherapy), by type of ICB (PD-1/PD-L1 or CTLA-4), and by indication, where data permitted. For both levels of analysis, regression scatter plots were used to present results. In the arm-level analyses, we evaluated the predictive values of PFS rate at 6 months and at 9 months relative to the OS rate at 18 months for all studies (various solid tumors) with a separate analysis limited to NSCLC-only studies. For each of these investigations, subanalyses were conducted to assess effects linked to type of therapy (single-agent ICBs vs both single-agent ICBs plus ICBs in combination with chemotherapy) and class of ICB (PD-1/PD-L1 vs CTLA-4). In the comparison-level analyses, we evaluated whether the ORR OR and the DCR OR could predict the OS HR in the pooled analysis as well as in the single-agent ICB analysis. In addition, we evaluated whether the PFS HR at 6 months could predict the OS HR in the pooled analysis as well as in the single-agent ICB analysis. In this analysis, data from patients treated with a combination of 2 ICBs were not included.

To determine surrogacy, we used the following threshold criteria (Kemp and Prasad 2017; Validity of surrogate endpoints in oncology Executive summary of rapid report A10-05, Version 1.1 2005): low correlation was indicated by *r* ≤ 0.7 (corresponding to *R*^2^ ≤ 0.49), medium strength correlation was indicated by *r* > 0.7 to r < 0.85 (corresponding to *R*^2^ > 0.49 to *R*^2^ < 0.72), and high correlation was indicated by *r* ≥ 0.85 (corresponding to *R*^2^ ≥ 0.72).

## Results

**Fig. 1:**
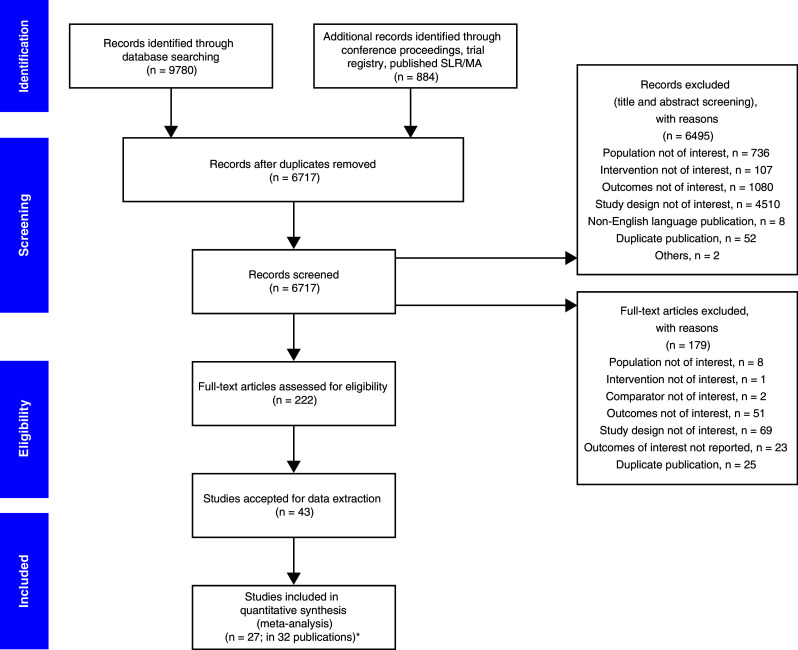
PRISMA flow diagram. Graphical representation of the flow of citations reviewed in the course of this systematic review, including number of records identified, included and excluded, and the reasons for exclusions. *Melanoma: CA184-022 (NCT00289640), CA184-025 (NCT00162123), CA184-004 (NCT00261365), CA184-013 (NCT00050102), E1608 (NCT01134614), NCT01740297, CheckMate 069 (NCT01927419), CheckMate 064 (NCT01783938), KEYNOTE-002 (NCT01704287), CA184-024 (NCT00324155), NCT00257205, CheckMate 066 (NCT01721772), KEYNOTE-006 (NCT01866319), CheckMate 037 (NCT01721746). NSCLC: CA184-041 (NCT00527735), POPLAR (NCT01903993), KEYNOTE-021 (NCT02039674), KEYNOTE-010 (NCT01905657), CheckMate 017 (NCT01642004), CheckMate 057 (NCT01673867), CheckMate 026 (NCT02041533), KEYNOTE-024 (NCT02142738), OAK (NCT02008227). UC: CheckMate 032 (NCT01928394), KEYNOTE-045 (NCT02256436). RCC: CA209-010 (NCT01354431). HNSCC: CheckMate 141 (NCT02105636). *HNSCC* head and neck squamous cell carcinoma,* MA* meta-analysis, *NSCLC* non-small cell lung cancer, *PRISMA* Preferred Reporting Items for Systematic Reviews and Meta-Analyses, *RCC* renal cell carcinoma, *SLR* systematic literature review, *UC* urothelial carcinoma

We identified 32 publications that met the inclusion criteria for a total of 27 RCTs involving 61 treatment arms and 10,300 patients (Fig. 1; Table 3). Most of these studies were conducted in patients with melanoma (52%; 14/27), followed by NSCLC (33%; 9/27); 2 trials were conducted in patients with UC, one study in patients with RCC, and one study in patients with HNSCC. Most studies (59%; 16/27) evaluated the efficacy of ICBs vs chemotherapy, and among these, 81% (13/16) investigated single-agent ICBs; 11% (3/27) of studies investigated ICBs in combination with chemotherapy. Ipilimumab monotherapy and nivolumab monotherapy were investigated in eight studies each (30%), pembrolizumab monotherapy in five studies (19%), atezolizumab monotherapy in two studies, and tremelimumab monotherapy in one study. Ipilimumab was tested as part of a combination regimen or a sequential therapy approach in nine studies, nivolumab-based combination regimens were assessed in two studies, and pembrolizumab-based combination regimens were assessed in one study. The analysis plan included assessments that stratified by PD-L1 expression of patient tumor samples, and while 48% (13/27) of studies reported PD-L1 expression status, the testing methods and thresholds for PD-L1 expression status were not uniform, thereby precluding a meaningful stratified analysis. From digitized Kaplan–Meier curves, 24 arms of virtual arm-level data were generated for PFS HRs at 6 and 9 months. Rates for PFS at 6 and 9 months and OS at 12 and 18 months were calculated from Kaplan–Meier curves of 11 RCTs.

**Table 3:** RCTs and related publications included in meta-analysis

Trial	Phase	Study design		Author	Journal or congress	Year
		ICB arm(s)	Comparator arm(s)			
Melanoma						
CA184-022 NCT00289640 (Wolchok et al. 2010)	2	Ipilimumab 0.3 mg/kg vs ipilimumab 3 mg/kg vs ipilimumab 10 mg/kg	NA	Wolchok JD	*Lancet*	2009
CA184-025 NCT00162123 (Thompson et al. 2012; Weber et al. 2009)	2	Ipilimumab + prophylactic budesonide vs ipilimumab + placebo	NA	Weber J	*Clin Cancer Res*	2009
				Thompson JA	*J Immunother*	2012
CA184-004 NCT00261365 (Hamid et al. 2011)	2	Ipilimumab 3 mg/kg vs ipilimumab 10 mg/kg	NA	Hamid O	*J Transl Med*	2011
MDX010-08 CA184-013 NCT00050102 (Hersh et al. 2011)	2	Ipilimumab vs ipilimumab + dacarbazine	NA	Hersh E	*Invest New Drugs*	2011
E1608 NCT01134614 (Hodi et al. 2014)	2	Ipilimumab + sargramostim vs ipilimumab	NA	Hodi FS	*JAMA*	2014
NCT01740297 (Chesney et al. 2016)	2	Ipilimumab + talimogene laherparepvec vs ipilimumab	NA	Chesney J	ESMO Annual Meeting	2016
CheckMate 069 NCT01927419 (Hodi et al. 2016a)	2	Nivolumab + ipilimumab vs ipilimumab	NA	Hodi FS	*Lancet Oncol*	2016
CheckMate 064 NCT01783938 (Weber et al. 2016b)	2	Nivolumab followed by ipilimumab vs Ipilimumab followed by nivolumab	NA	Weber JS	*Lancet Oncol*	2016
KEYNOTE-002 NCT01704287 (Hamid et al. 2016)	2	Pembrolizumab	Paclitaxel + carboplatin or paclitaxel or carboplatin or dacarbazine or oral temozolomide	Hamid O	ESMO Annual Meeting	2016
CA184-024 NCT00324155 (Maio et al. 2015; Robert et al. 2011)	3	Ipilimumab + dacarbazine	Dacarbazine + placebo	Robert C	*N Engl J Med*	2011
				Maio M	*J Clin Oncol*	2015
NCT00257205 (Ribas et al. 2013)	3	Tremelimumab	Temozolomide or dacarbazine	Ribas A	*J Clin Oncol*	2013
CheckMate 066 NCT01721772 (Robert et al. 2015a)	3	Nivolumab	Dacarbazine	Robert C	*N Engl J Med*	2015
KEYNOTE-006 NCT01866319 (Robert et al. 2015b)	3	Pembrolizumab vs ipilimumab	NA	Robert C	N Engl J Med	2015
CheckMate 037 NCT01721746 (Weber et al. 2016a)	3	Nivolumab	Dacarbazine or carboplatin + paclitaxel	Weber J	SMR Annual Meeting	2016
NSCLC						
CA184-041 NCT00527735 (Lynch et al. 2012)	2	Ipilimumab + paclitaxel + carboplatin	Paclitaxel + carboplatin + placebo	Lynch TJ	*J Clin Oncol*	2012
POPLAR NCT01903993 (Fehrenbacher et al. 2016; Smith et al. 2016)	2	Atezolizumab	Docetaxel	Fehrenbacher L	*Lancet*	2016
				Smith D	ASCO Annual Meeting	2016
KEYNOTE-021 NCT02039674 (Langer et al. 2016)	2	Pembrolizumab + pemetrexed + carboplatin	Pemetrexed + carboplatin	Langer CJ	*Lancet Oncol*	2016
KEYNOTE-010 NCT01905657 (Herbst et al. 2016)	2/3	Pembrolizumab 3 mg/kg q3wk or pembrolizumab 10 mg/kg q3wk	Docetaxel	Herbst RS	*Lancet*	2016
CheckMate 017 NCT01642004 (Brahmer et al. 2015; Spigel et al. 2015)	3	Nivolumab	Docetaxel	Brahmer J	*N Engl J Med*	2015
				Spigel DR	ASCO Annual Meeting	2015
CheckMate 057 NCT01673867 (Borghaei et al. 2015)	3	Nivolumab	Docetaxel	Borghaei H	*N Engl J Med*	2015
CheckMate 026 NCT02041533 (Socinski et al. 2016)	3	Nivolumab	Carboplatin + pemetrexed or Cisplatin + pemetrexed or Carboplatin + gemcitabine or Cisplatin + gemcitabine or Carboplatin + paclitaxel	Socinski M	ESMO Annual Meeting	2016
KEYNOTE-024 NCT02142738 (Reck et al. 2016)	3	Pembrolizumab	Carboplatin + pemetrexed or Cisplatin + pemetrexed or Carboplatin + gemcitabine or Cisplatin + gemcitabine or Carboplatin + paclitaxel	Reck M	*N Engl J Med*	2016
OAK NCT02008227 (Rittmeyer et al. 2017)	3	Atezolizumab	Docetaxel	Rittmeyer A	*Lancet*	2016
UC						
CheckMate 032 NCT01928394 (Rosenberg et al. 2016; Sharma et al. 2016)	1/2	Nivolumab vs Nivolumab + ipilimumab	NA	Sharma P	SITC Annual Meeting	2016
				Rosenberg JE	ESMO Annual Meeting	2016
KEYNOTE-045 NCT02256436 (Bellmunt et al. 2017)	3	Pembrolizumab	Paclitaxel or docetaxel or vinflunine	Bellmunt J	*N Engl J Med*	2017
RCC						
CA209-010 NCT01354431 (Motzer et al. 2015)	2	Nivolumab 0.3 mg/kg vs nivolumab 2 mg/kg vs nivolumab 10 mg/kg	NA	Motzer RJ	*J Clin Oncol*	2015
HNSCC						
CheckMate 141 NCT02105636 (Ferris et al. 2016)	3	Nivolumab	Methotrexate or docetaxel or cetuximab^a^	Ferris RL	*N Engl J Med*	2016

Details of clinical trials included in the meta-analysis organized by tumor type, along with related publications

*ASCO* American Society of Clinical Oncology, *ESMO* European Society for Medical Oncology, *HNSCC* head and neck squamous-cell carcinoma, *ICB* immune checkpoint blocker, *NA* not applicable, *q3w* every 3 weeks, *RCC* renal-cell carcinoma, *RCT* randomized, controlled trial, *SITC* Society for Immunotherapy of Cancer, *SMR* Society for Melanoma Research

^a^The proportion of patients who received cetuximab is fairly small, 15/121; therefore, the study is included in the analyses and the control arm is coded as “chemotherapy”

**Fig. 2:**
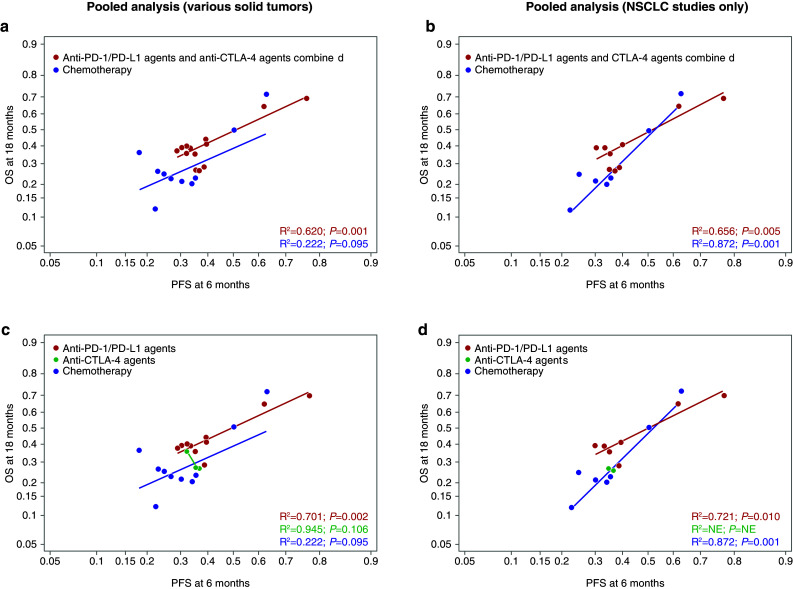
Arm-level analyses of the correlation between PFS at 6 months and OS in the pooled ICB studies. PFS rate at 6 months predicting OS rate at 18 months in the pooled analysis (**a**), in NSCLC-only studies included in the pooled analysis (**b**), in the pooled analysis stratified by class of ICB therapy (**c**), and in NSCLC-only studies included in the pooled analysis stratified by class of ICB therapy (**d**). *CTLA-4* cytotoxic T-lymphocyte-associated antigen-4, *ICB* immune checkpoint blocker,* NE* not estimable, *NSCLC* non-small cell lung cancer,* OS* overall survival, *PD-1* programmed cell death-1, *PD-L1* programmed cell death ligand-1, *PFS* progression-free survival

**Fig. 3:**
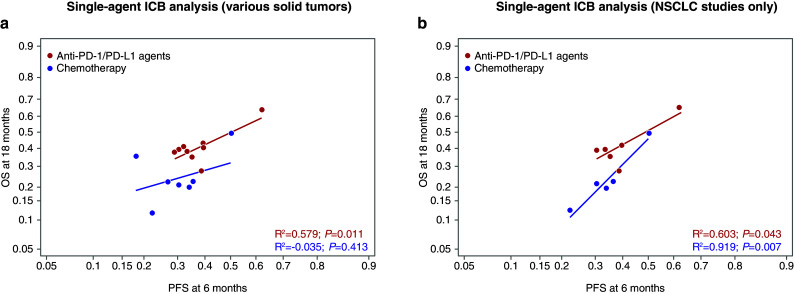
Arm-level analyses of the correlation between PFS and OS in the single-agent ICB studies. PFS rate at 6 months predicting OS rate at 18 months in the single-agent ICB analysis (**a**), and in NSCLC-only studies included in the single-agent ICB analysis (**b**). *CTLA-4* cytotoxic T-lymphocyte-associated antigen-4, *ICB* immune checkpoint blocker, *NSCLC* non-small-cell lung cancer, *OS* overall survival, *PD-1* programmed cell death-1, *PD-L1* programmed cell death ligand-1, *PFS* progression-free survival

**Fig. 4:**
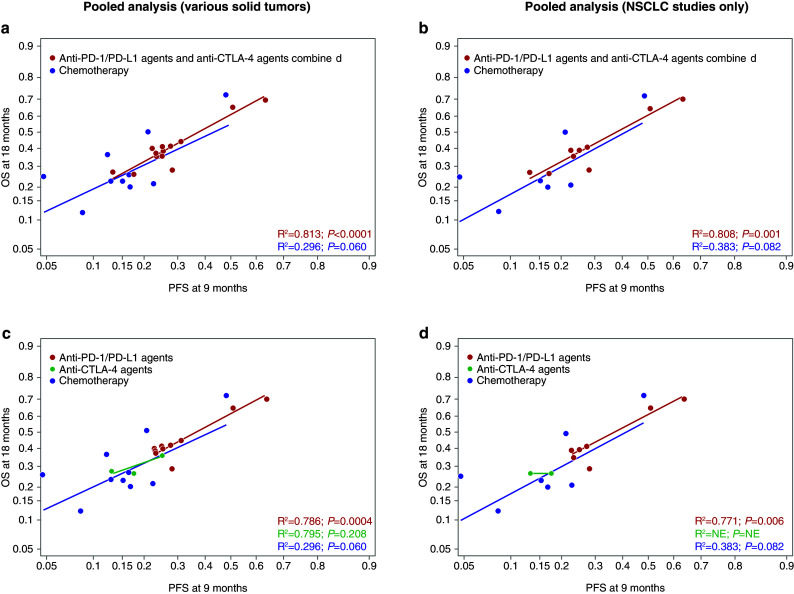
Arm-level analyses of the correlation between PFS at 9 months and OS in the pooled ICB studies. PFS rate at 9 months predicting OS rate at 18 months in the pooled analysis (**a**), in NSCLC-only studies included in the pooled analysis (**b**), in the pooled analysis stratified by class of ICB therapy (**c**), and in NSCLC-only studies included in the pooled analysis stratified by class of ICB therapy (**d**). *CTLA-4* cytotoxic T-lymphocyte-associated antigen-4, *ICB* immune checkpoint blocker, *NSCLC* non-small-cell lung cancer, *OS* overall survival, *PD-1* programmed cell death-1, *PD-L1* programmed cell death ligand-1, *PFS* progression-free survival

**Fig. 5:**
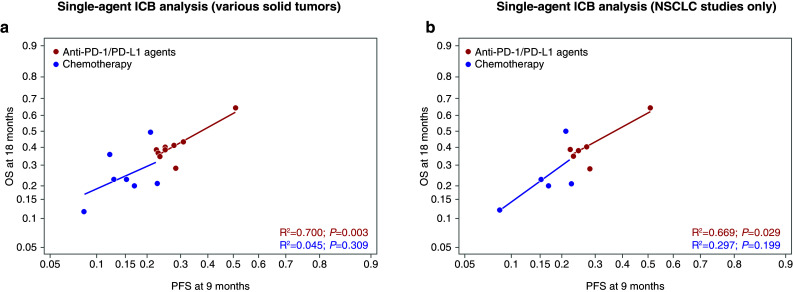
Arm-level analyses of the correlation between PFS at 9 months and OS in the single-agent ICB studies. PFS rate at 9 months predicting OS rate at 18 months in the single-agent ICB analysis (**a**), and in NSCLC-only studies included in the single-agent ICB analysis (**b**). *CTLA-4* cytotoxic T-lymphocyte-associated antigen-4, *ICB* immune checkpoint blocker, *NE* not estimable, *NSCLC* non-small-cell lung cancer, *OS* overall survival, *PD-1* programmed cell death-1, *PD-L1* programmed cell death ligand-1, *PFS* progression-free survival

Overall, the relationship between absolute effects for ICBs was similar to chemotherapy in that higher PFS rates at 6 months predicted higher OS rates at 18 months (Fig. 2). However, compared with chemotherapy arms, the ICB arms had a higher average OS rate for any given PFS rate (Fig. 2). The relationships between variables were the same in the pooled and in the single-agent ICB analyses (Figs. 2a, 3a). Results by type of ICB revealed stronger correlations for PD-1/PD-L1 than for CTLA-4 between potential surrogates and OS (Fig. 2c). Although there were very few anti-CTLA-4 studies, some analyses suggest that there may be a lower or nonexistent relationship at the absolute level between surrogates and OS in studies investigating antibodies against CTLA-4. This would need to be confirmed with a larger sample size. The relationship between PFS rates at 6 months and OS rates at 18 months was similar in studies conducted in various tumor types (Figs. 2a, 3a), as well as in those conducted in NSCLC only (Figs. 2b, 3b). For the NSCLC analysis, although the lines do cross, the slopes of the lines are not significantly different for the anti-PD-1/PD-L1 agents vs chemotherapy and indicate no distinct relationship particular to NSCLC patients. In addition to the correlation observed between 6-month PFS and 18-month OS, a similar correlation between 9-month PFS and 18-month OS was found (Figs. 4, 5).

**Fig. 6:**
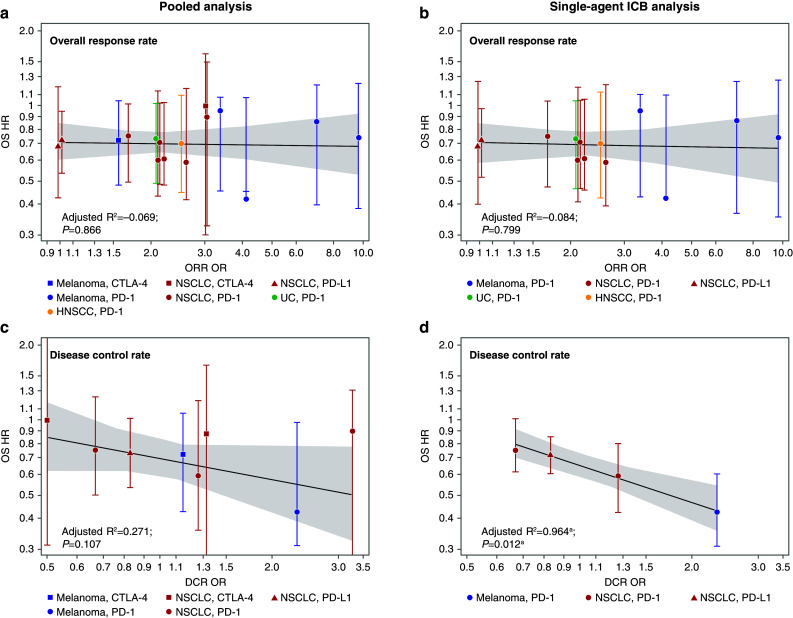
Comparison-level analyses of the correlation between ORR/DCR and OS. ORR and DCR odds ratio predicting OS hazard ratio in the pooled analysis (**a, c**, respectively), and in the single-agent ICB analysis (**b, d**, respectively). ^a^Please note this analysis is based on only 4 studies, hence, we cannot draw conclusions based on *R*^2^ and *P* values. *CTLA-4* cytotoxic T-lymphocyte-associated antigen-4, *DCR* disease control rate, *HNSCC* head and neck squamous cell carcinoma,* HR* hazard ratio,* ICB* immune checkpoint blocker,* NSCLC* non-small cell lung cancer,* OR* odds ratio,* ORR* objective response rate,* OS* overall survival,* PD-1* programmed cell death-1,* PD-L1* programmed cell death ligand-1,* UC* urothelial carcinoma

The comparison-level analysis shows that, across the included studies, treatment superiority on some surrogate endpoints is weakly predictive of treatment superiority on the final outcome (OS). There was largely a weak or a nonsignificant correlation between either ORR OR or DCR OR and OS HR and this held true even when the data were stratified by treatment type (Fig. 6). In the pooled analysis, there was no significant correlation between ORR OR and OS HR (adjusted *R*^2^ = − 0.069; *P* = 0.866; Fig. 6a). Likewise, the relationship between DCR OR and OS HR was not statistically significant (adjusted *R*^2^ = 0.271; *P* = 0.107; Fig. 6c). In the single-agent ICB analysis, there was no significant correlation between ORR OR and OS HR (adjusted *R*^2^ = − 0.084; *P* = 0.799; Fig. 6b), and the correlation between DCR OR and OS was statistically significant, although this DCR analysis was based on a limited number of studies (*n* = 4; adjusted *R*^2^ = 0.964; *P* = 0.012; Fig. 6d). The relationship between DCR OR and OS is statistically significant, but it is based on very limited data and the slope remains nearly flat, close to zero, making it of limited utility even were it to be confirmed with additional data.

**Fig. 7:**
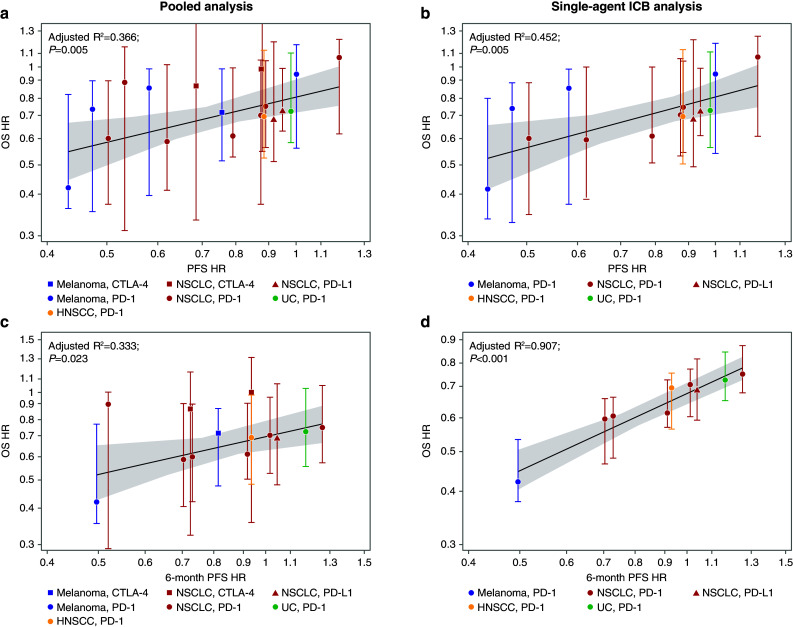
Comparison-level analyses of the correlation between PFS and OS. PFS hazard ratio predicting OS hazard ratio in the pooled analysis (**a**), as well as in the single-agent ICB analysis (**b**), and 6-month PFS HR predicting OS hazard ratio in the pooled analysis (**c**), as well as in the single-agent ICB analysis (**d**). *CTLA-4* cytotoxic T-lymphocyte-associated antigen-4, *HNSCC* head and neck squamous cell carcinoma,* HR* hazard ratio, *ICB* immune checkpoint blocker, *NSCLC* non-small cell lung cancer, *OS* overall survival, *PD-1* programmed cell death-1, *PD-L1* programmed cell death ligand-1, *PFS* progression-free survival, *UC* urothelial carcinoma

There was a weak to moderate correlation between the PFS HR and the OS HR (Fig. 7). In the pooled analysis, the PFS HR correlated weakly with OS HR (adjusted *R*^2^ = 0.366; *P* = 0.005; Fig. 7a) and this correlation remained consistent in the single-agent ICB analysis (adjusted *R*^2^ = 0.452; *P* = 0.005; Fig. 7b). The PFS HR at 6 months was highly predictive of OS HR in the single-agent ICB analysis (adjusted *R*^2^ = 0.907; *P* < 0.001; Fig. 7d), but was weakly predictive in the pooled analysis (adjusted *R*^2^ = 0.333; *P* = 0.023; Fig. 7c).

## Discussion

The arm-level analysis indicated that higher PFS rates at 6-month predicted better OS rates at 18 months regardless of therapy. The comparison-level analysis showed that, among anti-PD-1/PD-L1 studies, PFS was an imperfect surrogate (low-to-moderate correlation) for OS, whereas ORR was not correlated with OS. DCR was not correlated with OS in the pooled analysis, but was correlated with OS in the single-agent ICB analysis. The predictive value of PFS HR at 6 months for OS HR in the single-agent ICB analysis was the strongest. Unfortunately, though this correlation is statistically significant in the analysis, it has little clinical value. For the majority of included studies, the PFS HRs’ cluster around 0.9–1.0, indicating little to no treatment effect of single-agent ICBs on PFS compared with chemotherapy. This corresponds to an OS HR of ~ 0.7, indicating OS advantage for a single-agent ICB vs chemotherapy. Although the minimal impact of a single-agent ICB on PFS may still underestimate the OS benefit, in a registrational trial of a new ICB, it would be illogical to predict an OS benefit of a single-agent ICB by this standard, as it would imply that a finding of PFS of near 1.0 would yield an OS of 0.7, possibly strong enough to declare success.

In a recent meta-analysis of 25 RCTs including a total of 20,013 patients with metastatic NSCLC (only 6 trials involved ICBs), a moderate association was found between OS rate at 12 months and OS HR (*R*^2^ = 0.80) and a modest association was found between OS rate at 9 months and OS HR (*R*^2^ = 0.67) (Blumenthal et al. 2017). The meta-analysis from Blumenthal et al. also found modest associations between ORR at 6 months and PFS HR (*R*^2^ = 0.70), and PFS rate at 9 months and PFS HR (*R*^2^ = 0.62) (Blumenthal et al. 2017). Our study was not designed to investigate correlations between OS rate and OS HR, or between ORR rate and PFS HR, or between PFS rate and PFS HR. However, both studies analyzed correlations between PFS and OS HR, and between ORR and OS HR. According to the meta-analysis from Blumenthal et al., there were no associations between the ORR at 6 months and OS HR, or between PFS rate at 9 months and OS HR, by trial-level analysis (Blumenthal et al. 2017). Our study, which involved 27 RCTs, all of which included ICBs, also found no association between ORR and OS HR by trial-level analysis, but did find an association between PFS HR at 6 months and OS HR, which was limited to the single-agent ICB analysis (adjusted *R*^2^ = 0.907).

Although used in ICB clinical trials, RECIST 1.1 criteria may not be the best metric to determine the clinical benefit associated with ICBs (Bellmunt et al. 2017; Hodi et al. 2016a; Ribas et al. 2013; Rittmeyer et al. 2017; Robert et al. 2015b). Chemotherapeutic agents are cytotoxic and act directly on rapidly dividing tumor cells, so these agents can quickly shrink tumor size, translating into an antitumor response as determined by RECIST 1.1 criteria (Eisenhauer et al. 2009). However, this antitumor response may not be sustained over time, so that an initial PFS benefit may not translate into an OS benefit (Booth and Eisenhauer 2012; Gatzemeier et al. 2000). In contrast, ICBs act on the immune system, whereby tumor-infiltrating lymphocytes and infiltration by other immune cells may lead to an initial increase in tumor size. This phenomenon is referred to as “pseudoprogression”, because by RECIST 1.1 standards, the apparent increase in tumor size indicates disease progression (Wolchok et al. 2009). With ICBs, the size increase may not be an increase in tumor burden, but rather an artifact of the inflammatory response that can be followed by subsequent tumor shrinkage, translating into a durable antitumor response (Hersh et al. 2011; Hodi et al. 2016b; Seymour et al. 2017). To better assess antitumor response associated with ICBs, the iRECIST criteria have been developed, which modify RECIST 1.1 criteria to account for unusual patterns of immune-based responses observed with ICBs (Seymour et al. 2017).

As novel ICB-based combination approaches are being evaluated in clinical trials, alternative endpoints that may fully capture the potential benefit associated with ICBs are being explored and validated (Checkpoint Inhibitors Spur Changes in Trial Design 2017). Therefore, in the near future, analyses of correlations between OS and novel endpoints may be possible. Potential endpoints for consideration may include classical clinical endpoints defined by the novel irRC/irRECIST/iRECIST criteria (Nishino et al. 2013; Seymour et al. 2017; Wolchok et al. 2009), which allow for progression prior to response, or newer endpoints defined by these criteria, such as durable response rate or sustained reduction or stability in overall tumor burden (Checkpoint Inhibitors Spur Changes in Trial Design 2017; Kaufman et al. 2017).

### Study limitations

This study used only aggregate summary data from published studies and no patient-level data; therefore, we cannot necessarily assume that any statistical association observed between group-level variables may be translated to individual-level associations for analyses at the trial level. Therefore, our findings cannot be used to predict any outcome at the individual level. Analyses at arm level are limited by the inherent associations between different clinical endpoints and outcomes assessed. The relationship between potential clinical surrogate endpoints and OS may be further obscured by study crossover, wherein patients are allowed to switch from the control arm to the active treatment arm, thereby altering the disease course (Flaherty et al. 2014); and the extent of crossover is not always reported in the published studies, nor is the cross-over unadjusted/adjusted OS. In addition, due to the paucity of data, stratified analyses by indication or treatment type have limited power in detecting substantive relationships. Another limitation of this study regards the analysis conducted on DCR, given the fact that the duration requirement for stable disease, as part of the definition of DCR, differs across trials. Furthermore, this analysis is based on published data for ICBs that ultimately gained FDA approval; therefore, it remains uncertain how the surrogate endpoints assessed correlate to OS in the context of other ICBs not proven to impact OS. There are several studies underway that, once completed, will provide additional data for analysis, and may result in supporting more robust associations.

## Conclusions

This study and previous meta-analyses have failed to identify a clinical endpoint that is suitable as a surrogate for OS in studies involving ICBs, with PFS HR at 6 months being a moderately strong predictor of OS for studies involving single-agent ICBs. Although identification of baseline gene signatures predictive of response has gained some traction (e.g., tumor mutational burden or interferon gamma gene signatures), there are few publications that attempt to identify markers (biologic, radiologic, or otherwise) that correlate with OS and can be measured at early timepoints after the onset of ICB therapy. As none of classical clinical endpoints used in oncology trials was found as potential surrogate for OS, it is of paramount importance that efforts to identify novel surrogates for efficacy be supported and encouraged in academia and in the biotech/pharma industry to expedite the development of life-saving drugs.

## Data Availability

The data sets used and/or analyzed during the current study are available from the corresponding author on reasonable request.

## Abbreviations

AHNS: American Head and Neck Society
ASCO: American Society of Clinical Oncology
CTLA-4: Cytotoxic T-lymphocyte-associated antigen-4
DCR: Disease control rate
ELCC: European Lung Cancer Conference
ESMO: European Society for Medical Oncology
FDA: Food and Drug Administration
HNSCC: Head and neck squamous-cell carcinoma
HR(s): Hazard ratio(s)
ICB: Immune checkpoint blocker
irRC: Immune-related response criteria
ITT: Intent to treat
MA: Meta-analysis
NA: Not applicable
NE: Not estimable
NSCLC: Non-small-cell lung cancer
OR(s): Odds ratio(s)
ORR: Overall response rate
OS: Overall survival
PD-1: Programmed cell death-1
PD-L1: Programmed cell death ligand-1
PFS: Progression-free survival
PRISMA: Preferred reporting items for systematic reviews and meta-analyses
q3w: Every 3 weeks
RCC: Renal-cell carcinoma
RCTs: Randomized controlled trials
RECIST: Response evaluation criteria in solid tumors
SITC: Society for Immunotherapy of Cancer
SLR: Systematic literature review
SMR: Society for Melanoma Research
UC: Urothelial carcinoma
WHO: World Health Organization
